# Polymer Melt Stability Monitoring in Injection Moulding Using LSTM-Based Time-Series Models

**DOI:** 10.3390/polym18010032

**Published:** 2025-12-23

**Authors:** Pedro Costa, Sílvio Priem Mendes, Paulo Loureiro

**Affiliations:** 1 School of Technology and Management, Polytechnic University of Leiria, 2411-901 Leiria, Portugal; 2220129@my.ipleiria.pt (P.C.); smendes@ipleiria.pt (S.P.M.); 2 Computer Science and Communication Research Centre, School of Technology and Management, Polytechnic University of Leiria, 2411-901 Leiria, Portugal

**Keywords:** defect prediction, LSTM, injection moulding, real production data, data-driven solution

## Abstract

This work presents a data-driven framework for early detection of polymer melt instability in industrial injection moulding using Long Short-Term Memory (LSTM) time-series models. The study uses six months of continuous production data comprising approximately 280,000 injection cycles collected from a fully operational thermoplastic injection line. Because melt behaviour evolves gradually and conventional threshold-based monitoring often fails to capture these transitions, the proposed approach models temporal patterns in torque, pressure, temperature, and rheology to identify drift conditions that precede quality degradation. A physically informed labelling strategy enables supervised learning even with sparse defect annotations by defining volatile zones as short time windows preceding operator-identified non-conforming parts, allowing the model to recognise instability windows minutes before defects emerge. The framework is designed for deployment on standard machine signals without requiring additional sensors, supporting proactive process adjustments, improved stability, and reduced scrap in injection moulding environments. These findings demonstrate the potential of temporal deep-learning models to enhance real-time monitoring and contribute to more robust and adaptive manufacturing operations.

## 1. Introduction

Injection moulding is one of the most widely used manufacturing processes for producing thermoplastic parts, especially in high-volume sectors such as automotive, consumer goods, and medical devices. Although the process is robust and largely repeatable, the quality of moulded components is highly sensitive to the thermo-rheological behaviour of the polymer melt. Small variations in melt temperature, shear rate, or residence time can alter viscosity and flow dynamics, potentially leading to defects such as short shots, warpage, sink marks, weld-line weakness, or incomplete filling. Because these deviations often evolve gradually over several cycles, early detection is essential for preventing drift toward unstable operating conditions.

Conventional process-monitoring systems typically rely on predefined thresholds or rule-based logic. While effective for detecting abrupt failures, such static approaches are unable to capture the nonlinear and time-dependent relationships that characterise melt behaviour. Minor shifts in torque, pressure, or thermal load may precede visible defects by several minutes, yet remain undetected by threshold-based models. This has motivated the adoption of machine-learning (ML) approaches capable of learning complex patterns directly from sensor data.

Recent research has explored unsupervised anomaly detection, autoencoder-based reconstruction, and generative modelling for identifying deviations in injection moulding processes. However, many existing approaches treat cycle data as independent observations, disregarding the sequential nature of melt dynamics. Polymer viscosity is inherently time-dependent, influenced by accumulated thermal fluctuations, shear-thinning effects, and barrel-residence conditions. As a result, models that analyse cycles in isolation fail to capture the gradual transitions that typically precede melt instability.

Long Short-Term Memory (LSTM) neural networks offer a suitable framework for modelling these temporal dependencies. By learning patterns across multiple successive cycles, LSTMs can detect evolving melt-flow deviations that static or cycle-independent methods are unable to identify. Their ability to integrate sequential sensory information makes them particularly effective in industrial scenarios where slow drift, rather than abrupt anomalies, is the dominant precursor to quality degradation.

In this context, the present work proposes an LSTM-based monitoring framework designed to detect early-stage polymer melt instability using real production data from a fully operational industrial injection moulding line. The study addresses practical challenges encountered in real environments, including limited defect labels, correlated sensor measurements, ambiguous transitional periods, and the need for physically interpretable outputs.

While LSTM architectures have been widely used for time-series modelling, the novelty of this work lies in their application to real industrial injection moulding data using physically informed volatility windows, enabling early detection of gradual rheological drift under production constraints.

### Research Gap and Main Contributions

Despite progress in data-driven monitoring for injection moulding, a clear research gap remains due to several persistent limitations:

1. Static monitoring approaches using threshold-based or cycle-independent methods cannot capture the gradual temporal drift that characterises melt instability.
2. Many anomaly-detection methods treat cycles as independent observations, overlooking the temporal dependencies that precede instability.
3. Supervised learning approaches typically require extensive defect annotations, which are limited in industrial environments.
4. Transitional “volatile” periods are rarely labelled, complicating reliable supervised model training.
5. Existing frameworks do not integrate temporal modelling with sparse labels while relying solely on standard machine signals available in industrial settings.

This work addresses these gaps by proposing an LSTM-based framework that learns sequence-level melt-behaviour patterns and enables early detection of instability using routinely collected production data.

The main contributions of this work are as follows:

1. A data-processing pipeline integrating sensor interpretation, volatility-zone labelling, correlation-based pruning, and physically informed feature engineering.
2. A comparative evaluation of torque-only, correlation-pruned, and PCA-derived feature sets to assess trade-offs between feature compactness and predictive performance.
3. A systematic study of short- and extended-horizon LSTM architectures for real-time prediction of defect-prone operating windows.
4. A physically grounded interpretation of learned temporal patterns, connecting model behaviour with known thermo-rheological mechanisms of polymer melt.

Overall, this study demonstrates that temporal deep-learning models can identify early rheological drift in injection moulding, providing a practical path toward real-time monitoring and proactive process control.

## 2. Related Work

Anomaly detection in industrial environments has been extensively studied due to the growing availability of high-frequency sensor data and the demand for predictive maintenance and quality assurance. Traditional anomaly detection methods for time-series data are well established in the literature. Gupta et al. [1] provided one of the earliest comprehensive surveys on temporal outlier detection, discussing statistical and distance-based approaches. More recent work by Pang et al. [2] reviewed deep learning techniques for anomaly detection, emphasising the advantages of representation learning for complex temporal structures. Bardos et al. [3] compared supervised and unsupervised anomaly-detection strategies in industrial settings, highlighting the importance of explainability when deploying models in production environments.

Within the injection moulding domain, early approaches focused on unsupervised learning. Schiffers and Honysz [4] used clustering and reconstruction-error methods to detect irregularities in cycle data, while Wang et al. [5] introduced generative adversarial networks (GANs) as a means of modelling normal process behaviour. Hybrid solutions combining statistical process control with deep autoencoders have also been explored. Tayalati et al. [6] demonstrated a hybrid DL–SPC framework for anomaly detection in injection moulding, showing that deep learning can effectively complement conventional quality-control strategies. More recent studies by Kim et al. [7] and Tayalati et al. [8] expanded this concept by integrating multimodal deep learning and digital twin systems for process monitoring and predictive fault detection in plastic injection moulding.

Recent publications in *Polymers* highlight a growing interest in integrating machine learning with polymer-processing science. Liou et al. [9] and Tseng et al. [10] demonstrated that nozzle-pressure and screw-position signals can be used to optimise injection moulding process parameters through adaptive control methods. Similarly, Fernández et al. [11] proposed a predictive methodology for part-quality assessment, illustrating the potential of ML algorithms to improve defect detection. Wenzel et al. [12] analysed hybrid machine learning approaches for surrogate modelling of part shrinkage, emphasising the role of physical–data-driven models in polymer flow simulation. Complementary research by Ding et al. [13] introduced deep-learning-based soft sensors for predicting part weight, while Raimi and Lee [14] applied LSTM architectures to forecast demoulding force, both enhancing process monitoring accuracy.

Beyond injection moulding, machine learning has also been applied to other polymer-processing workflows. Munir et al. [15] explored interpretable ML techniques for monitoring extrusion-induced degradation in PLA materials, demonstrating how rheological changes can be captured from sensor data. Bielenberg et al. [16] reviewed the evolution of adaptive control in injection moulding, documenting the transition from manual to automated process regulation. Krantz et al. [17] applied ML-based control strategies to moulding of recycled polypropylene, illustrating how data-driven models can handle material variability, a persistent challenge in polymer processing. Related studies have also explored predictive control approaches: Ren et al. [18] proposed an LSTM-based framework for injection velocity regulation, while Selvaraj and Raj [19] provided a broad review of machine learning models in injection moulding machines. Furthermore, Cao et al. [20] presented a comprehensive review on process-control evolution, highlighting deep learning’s growing impact in the field.

Although LSTM and transformer-based architectures have been applied in industrial monitoring contexts, most existing works focus on detecting abrupt anomalies or cycle-independent deviations rather than modelling gradual rheological drift [4,5,6]. These approaches also lack mechanisms to incorporate domain-specific melt-behaviour transitions, such as viscosity evolution and torque accumulation, that develop over multiple cycles in injection molding [9,10,15]. Furthermore, published LSTM-based studies seldom address the constraints of real production environments, including sparse defect labels and ambiguous transitional zones [3,11,17], limiting their applicability to the problem addressed in this work.

Collectively, these works demonstrate strong progress in the application of machine learning to process monitoring and polymer manufacturing. However, most existing approaches either rely on static unsupervised models that fail to capture temporal dependencies or require extensive defect labels that are rarely available in industrial datasets. Moreover, although recent studies explore sensor-based monitoring, few explicitly connect machine-learning predictions with the thermo-rheological behaviour of polymer melts. This gap motivates the use of LSTM-based time-series models capable of learning gradual drifts in melt viscosity, pressure evolution, and torque dynamics, phenomena that precede unstable moulding conditions.

## 3. Data and Modelling Framework

This section details the methodological framework adopted to build a reliable, physically interpretable monitoring system for polymer melt stability. The workflow combines domain-driven feature understanding with systematic data preparation and sequential modelling. First, we examine how each sensor signal relates to the underlying thermo-rheological mechanisms governing melt behaviour. We then describe the data-processing pipeline, feature-selection strategies, and the architecture of the LSTM models used for anomaly prediction. Together, these steps establish the foundation for a modelling approach that is both data-driven and consistent with polymer-process physics.

### 3.1. Polymer-Process Interpretation of Features

Understanding the physical meaning of the monitored variables is essential to explain how sensor fluctuations reflect the behaviour of the polymer melt during injection moulding. While the LSTM model captures complex temporal dependencies automatically, linking each feature to the underlying thermo-rheological mechanisms of the polymer provides a material-science justification for the detected patterns.

#### 3.1.1. Torque (Mm and Ms) as Indicators of Melt Rheology

The torque values associated with screw rotation (Ms—medium torque and Mm—maximum torque) indirectly represent the resistance of the polymer melt to shear flow. Progressive increases in torque typically indicate rising melt viscosity caused by thermal degradation, insufficient heating, or non-uniform temperature distribution along the barrel. Such rheological instabilities affect filling behaviour, packing pressure, and melt homogeneity and are common precursors to defects such as short shots, weld lines, or incomplete filling. The clear statistical separation of these torque features between volatile and non-volatile zones shows their high sensitivity to rheological stability.

#### 3.1.2. Viscosity (Derived Feature) as a Direct Proxy of Melt Condition

The engineered viscosity feature combines injection peak pressure, filling time, and the machine’s intensification ratio, providing a physically grounded approximation of the mechanical effort required to drive the melt into the cavity. Because polymer viscosity is strongly dependent on temperature, shear rate, and thermal history, this composite feature effectively reflects the real melt state. Deviations in viscosity commonly arise minutes before defect-prone periods, supporting the idea that melt instability is a primary mechanism behind the observed “volatile zones”.

#### 3.1.3. Temperature-Related Effects and Thermal Stability of the Melt

Although several temperature features were removed due to low variance, small thermal fluctuations, particularly during restarts or transient phases, can lead to temperature gradients in the melt. These gradients directly influence viscosity, pressure, and shear behaviour and can trigger subtle process instabilities. These thermal effects often propagate to torque and pressure readings before any quality deterioration is detectable in the produced parts.

#### 3.1.4. Injection Pressure and Flow-Front Development

Injection pressure reflects the resistance of the melt during cavity filling. Slight oscillations may arise from material degradation, changes in internal friction, fluctuations in screw load, or variations in the runner and gating system. In industrial practice, drops or spikes in injection pressure preceding defective cycles are consistent with undesired changes in melt flow-front dynamics and packing behaviour, highlighting the relevance of pressure signals in feature selection.

#### 3.1.5. Link Between Physical Mechanisms and LSTM Detection

The superior performance of the LSTM model suggests that process instabilities do not manifest solely in the absolute values of the monitored features but rather in their temporal evolution. Slow drift in viscosity, gradual accumulation of torque, micro-fluctuations in pressure, and temperature-related transitions after production stops are all behaviours consistent with known polymer rheological mechanisms, such as viscosity–temperature coupling, progressive thermal degradation, and shear-thinning dynamics. These temporal patterns justify why sequential modelling is essential for identifying defect-prone periods in industrial injection moulding.

### 3.2. Data Processing

Raw sensor data was provided by a leading company in mould injection systems for thermoplastic automotive parts. The dataset spans six months of continuous production, operating 24/7 with brief maintenance breaks, captured from a single injection machine that produces two pieces per cycle (approximately 55 s per cycle). Data was recorded from 264 sensors, each providing one reading per cycle on variables such as temperature, pressure, torque, stage time, and force.

The data processing workflow began with an extensive exploration phase, combining statistical analyses with insights from the company’s process engineer. Steps included label cleaning, handling missing values (none detected), removing duplicate features (redundant cycle counter), feature reduction from 264 to 47 signals, data correction near production stops (remove 50 points after ≥2-min gaps), engineering a Viscosity feature (Viscosity=PVs×ZSx×8.67), and standardisation. This engineered viscosity feature reflects standard industrial practice in the case-study environment, where peak injection pressure (PVs) and screw stroke time (ZSx) are combined with the machine-specific intensification ratio (8.67) to approximate relative melt-flow resistance. This formulation was validated with process engineers and provides a reliable proxy for tracking viscosity-related behaviour in the absence of direct rheological measurements.

Production stops introduce transient thermal and mechanical effects that temporarily alter melt behaviour, causing the first cycles after a restart to deviate significantly from steady-state conditions. To prevent these outlier patterns from contaminating model training, we removed the 50 cycles following any interruption of ≥2 min. This interval reflects the time required for the machine to return to stable processing conditions, based on process-engineering knowledge and empirical testing. Although adjustable, this value was selected as the most representative threshold for restoring reliable sensor behaviour in the industrial setting studied.

### 3.3. Feature Selection

**Figure 1:**
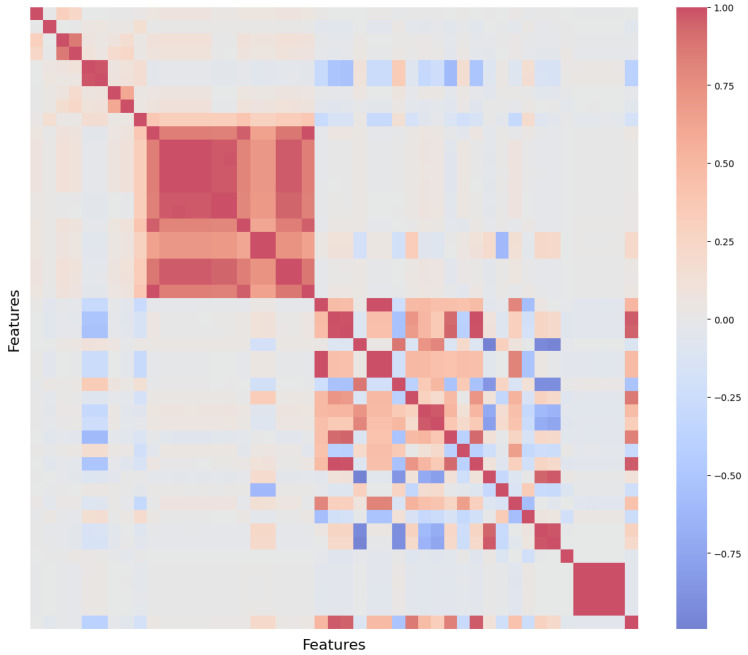
Pearson correlation matrix of the selected sensor signals after initial feature filtering, highlighting strong linear dependencies among process variables.

**Figure 2:**
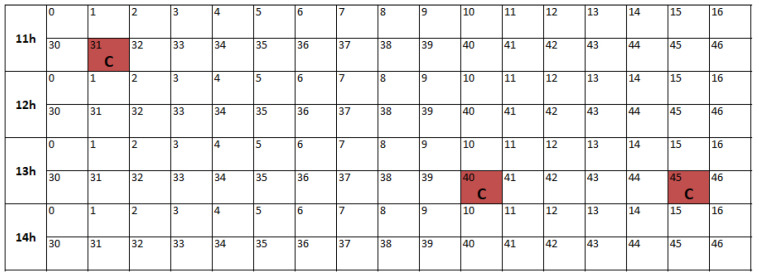
Excerpt of the operator fill-in form used to record non-conforming (C) parts and production events, serving as the basis for defining volatile zones.

**Figure 3:**
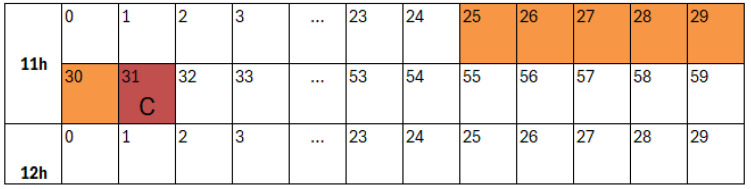
Example of a volatile zone defined as a short time window preceding an operator-identified non-conforming part, illustrating the temporal labelling strategy.

**Figure 4:**
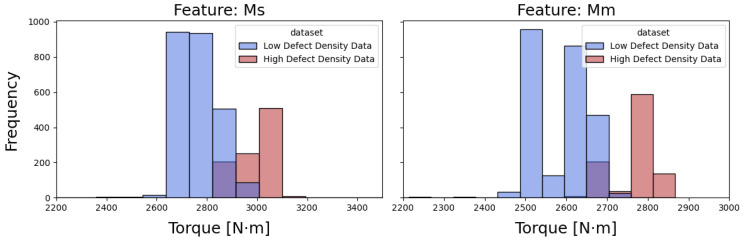
Distribution of medium torque (Ms) and maximum torque (Mm) values for stable and volatile operating conditions, showing their discriminative power for melt-instability detection.

Feature selection combined statistical analysis, domain expertise, and dimensionality-reduction techniques to obtain a compact and physically meaningful set of inputs for the LSTM models. From the original 264 recorded signals, preliminary filtering removed duplicate, near-constant, or operationally irrelevant variables, reducing the dataset to 47 candidate features. Three complementary strategies were applied thereafter: (i) group-wise Principal Component Analysis (PCA), (ii) correlation filtering, and (iii) domain-driven torque-feature selection. Figure 1, Figure 2, Figure 3 and Figure 4 illustrate the key steps of this process.

#### 3.3.1. Group-Wise PCA: Latent Physical Structure

PCA was applied to groups of sensors measuring related physical quantities, including mould temperatures, hydraulic pressures, screw loads, and stage durations. The strong internal correlations observed in the correlation matrix (Figure 1) indicated that many sensors exhibited similar behaviour within each group.

Across temperature, pressure, and timing sensors, the first principal component (PC1) consistently explained more than 95% of the variance, showing that each group was governed by a dominant physical factor:

- Temperature group. PC1 described the global thermal state of the moulding system. Because individual thermocouples differed only by small offsets, their collective behaviour was effectively one-dimensional. This justified reducing 14 temperature sensors to 6 PCA-derived components.
- Pressure and injection-dynamics group. PC1 captured the overall resistance of the melt during filling and packing, with high loadings on peak pressure, holding pressure, and screw force. PC2 represented variations in the shape and timing of the pressure rise, corresponding to transient instabilities in cavity filling.
- Torque and plastification group. Although torque features were ultimately retained in their original form, PCA indicated that PC1 corresponded to melt-viscosity variation: increases in torque and plastification time loaded heavily on this component, reflecting insufficient melting or local thermal imbalance.
- Cycle-time and operational group. PCA separated the normal production rhythm (PC1) from sporadic operational disturbances (PC2), such as robotic delays or operator intervention, enabling non-rheological influences to be isolated.

Overall, the PCA results revealed that melt stability is governed by a small number of latent physical mechanisms: effective viscosity, global thermal state, filling resistance, and operational variability, providing a compact, interpretable representation for modelling.

#### 3.3.2. Correlation Filtering

A Pearson correlation threshold of 0.90 was applied to remove redundant features that carried overlapping information. When two variables were strongly correlated, the feature with lower interpretability or engineering relevance was discarded. The correlation structure guiding this pruning process is shown in Figure 1.

#### 3.3.3. Physically Motivated Torque Selection

Operator-provided defect annotations were analysed using the fill-in form shown in Figure 2. These annotations defined the volatile windows preceding non-conforming parts, with an example illustrated in Figure 3. Among all monitored signals, the medium and peak torque values (Ms and Mm) showed the clearest distributional separation between volatile and non-volatile periods. This behaviour, highlighted in Figure 4, reflects the strong dependence of torque on melt viscosity and shear resistance.

Because torque responds sensitively to rheological drift minutes before defects appear, Ms and Mm were retained as standalone features and evaluated as a minimalistic two-feature baseline.

#### 3.3.4. Final Feature Sets

Based on these steps, the following three feature sets were constructed:

1. Double Torque (2 features): Ms and Mm;
2. Correlation-Pruned Set (14 features): Selected using the 0.90 correlation threshold and domain considerations;
3. Explained-Variance Set (33 features): PCA-reduced and domain-filtered components representing the main latent physical mechanisms.

These complementary feature sets enabled systematic evaluation of the trade-off between feature compactness, physical interpretability, and predictive performance in the subsequent LSTM modelling.

### 3.4. LSTM Architecture and Prediction Framework

The task is formulated as binary classification with a sliding window: inputs are sequences of 50 consecutive sensor readings; the label indicates whether the subsequent horizon contains a volatile zone. Two prediction strategies were tested: sequence-to-time step (predict the immediate next step) and sequence-to-sequence (predict whether any of the next *k* steps is volatile). The architecture uses a single LSTM layer (32 units, tanh), a dropout layer (rate 0.3), and a sigmoid output. Models were trained up to 50 epochs (batch size 32), with the Adam optimiser (learning rate 0.001), early stopping (patience 5), and class weights inversely proportional to class frequencies. Metrics include precision, recall, F1-score, and AUC.

## 4. Results and Discussion

This section presents the performance of the proposed LSTM models across different feature sets and prediction horizons. We begin with the sequence-to-time-step results, which showed the strongest and most consistent behaviour.

### Short-Horizon LSTM Prediction Results

This section presents the evaluation of all LSTM configurations under six experimental setups, combining three feature-selection strategies with two prediction horizons. The feature groups tested were (i) Double Torque (2 features), (ii) Pearson Correlation (14 features), and (iii) Explained Variance (33 features). All models were trained using sliding windows of 50 cycles and evaluated for both next-step (sequence-to-time-step) prediction and multi-step (sequence-to-sequence) prediction over a defect-prone horizon of seven cycles. Given the strong class imbalance present in the dataset, the F1-score was adopted as the primary metric for assessing anomaly detection performance.

A. Next-Step Instability Detection.

The sequence-to-time-step formulation yielded the strongest and most consistent results across all experiments. Although this approach predicts only the next time step, it effectively identifies the subtle temporal deviations that precede volatile operating conditions. As shown in Table 1, the two-feature torque-based model provided strong performance with a macro-average F1-score of 0.94, demonstrating that even minimal, but rheologically meaningful, feature sets are sufficient for accurate predictions.

Similarly, the 33-feature explained-variance set (see Table 2) achieved a macro-average F1-score of 0.95, with anomaly-class F1 around 0.91 and normal-class F1 around 0.96.

The identical performance values reported in Table 1 and Table 2 show that, for next-step prediction, torque features already capture the dominant discriminative information, and adding explained-variance features does not provide additional predictive benefit beyond torque-based inputs.

The Pearson 14-feature dataset also performed competitively (see Table 3), achieving a macro-average F1-score of 0.93, despite slightly lower anomaly-class recall.

These observations confirm that modelling short-horizon dynamics is an effective strategy for capturing drift conditions in injection moulding, where instability typically develops gradually rather than abruptly.

B. Polymer-Science Interpretation of Model Behaviour.

The predictive patterns learned by the LSTM model align closely with established thermo-rheological behaviour of polymer melts. Volatile periods in the dataset were preceded by slow drifts in melt viscosity and progressive increases in screw torque. Such behaviour is consistent with fluctuating shear conditions, insufficient or uneven plastification, and transient thermal imbalances within the barrel. These rheological deviations typically manifest before visible defects occur, such as incomplete filling or the formation of weld lines, explaining why the LSTM is able to anticipate instability windows several minutes before they become critical.

The model also showed high sensitivity to variations in injection pressure and filling time. Both parameters are directly tied to the resistance of the melt during cavity filling. Minor deviations in these signals often indicate changes in melt temperature, shear rate, or local viscosity. The model’s reliance on these variables demonstrates its ability to infer the physical state of the melt through patterns embedded in machine signals, even when explicit thermal measurements present limited variability.

Together, these findings show that the LSTM is not merely detecting statistical anomalies; it is identifying physically meaningful precursors to rheological instability.

C. Evidence of Non–Thermo-Rheological Influences.

While the LSTM models predominantly captured behaviour consistent with the thermo-rheological characteristics of polymer melts, several observations indicate that non-rheological factors also contributed to the predictions.

1. Mechanical torque effects: Although screw torque is strongly related to melt viscosity, it is also influenced by mechanical phenomena such as screw-drive friction, lubrication variability, servo-motor dynamics, or equipment wear. The strong performance of the two-feature torque configuration suggests that the LSTM may be responding to a mixture of mechanical and rheological signals.
2. Indirect detection of thermal variations: Due to the low variance in recorded melt and barrel temperatures, these features contributed minimally to the learning process. As a result, the model inferred thermal instabilities indirectly through pressure and torque patterns. Any thermal drift not reflected in these secondary features would not be detected directly.
3. Operational sources of variability: Occasional cycle-time irregularities, potentially caused by downstream robotics, conveyor delays, or operator intervention, also influenced anomaly predictions. These fluctuations are operational rather than rheological, indicating that the models capture process-level disturbances beyond melt behaviour.
4. Label ambiguity: The use of a seven-cycle volatile window introduced ambiguous training examples, particularly for sequence-to-sequence prediction. When a sequence containing both stable and unstable behaviour is labelled entirely as volatile, the discriminative signal becomes diluted, complicating the model’s ability to distinguish true melt-instability patterns from benign variations.

These observations reflect the realistic complexity of industrial data, in which mechanical, operational, and rheological effects interact simultaneously. While the model effectively anticipates melt-instability events, its predictions incorporate a combination of polymer-process signatures and machine-related behaviours, which is typical in real manufacturing environments.

D. Sequence-to-Sequence Prediction.

The sequence-to-sequence approach delivered lower accuracy than the single-step models. As reported in Table 4, Table 5 and Table 6, macro-average F1-scores plateaued around 0.82–0.83 across all feature groups, with anomaly-class recall significantly reduced.

This behaviour is largely attributable to ambiguous training labels caused by the 7-cycle volatile window definition.

E. Cross-Configuration Comparison.

A global comparison of all models, summarised in Table 7, highlights the superiority of the sequence-to-time-step LSTM configurations, which achieved the highest AUC values (0.960–0.975), with the 33-feature model performing best. These models consistently outperformed the sequence-to-sequence configurations, which reached lower AUC values in the range of 0.89–0.91 due to the inherent label ambiguity introduced by multi-step volatile windows. Overall, the results confirm that short-horizon temporal modelling provides the most reliable discrimination between stable and unstable melt-flow conditions in industrial injection moulding.

Although the primary focus of this work is on modelling melt-instability dynamics, it is worth noting the practical considerations that support deployment in industrial edge environments. The proposed LSTM models operate on a compact set of routinely collected machine signals, require modest computational resources due to their single-layer architecture, and do not rely on high-dimensional temporal reconstructions or large memory buffers. These characteristics make the approach compatible with typical on-machine controllers or embedded monitoring units, where processing power and latency constraints must be respected. While a full edge-deployment study is beyond the scope of this work, these properties indicate that the framework is suitable for integration into real-time monitoring pipelines in industrial settings.

Overall, model performance is affected by class imbalance, label ambiguity arising from volatile windows, and sensitivity to operational disturbances, reflecting the inherent complexity of real industrial injection moulding processes.

## 5. Conclusions and Future Work

This work presented a data-driven framework for monitoring polymer melt stability in injection moulding using Long Short-Term Memory (LSTM) neural networks. The approach was evaluated on several months of real industrial production data, incorporating routinely collected machine signals such as injection pressure, screw torque, screw position, and engineered viscosity indicators. Across all configurations, short-horizon sequence-to-time-step LSTM models consistently delivered the strongest performance, achieving F1-scores of 0.94–0.95 and AUC values above 0.97, confirming their suitability for identifying early signs of process drift.

The learned temporal patterns align closely with established thermo-rheological behaviour of polymer melts. Gradual viscosity drift, torque increases during plasticisation, and subtle injection-pressure fluctuations were identified as robust precursors to melt instability. These findings indicate that the LSTM models capture physically meaningful signals rather than purely statistical irregularities. At the same time, some sensitivity to non-rheological factors, such as mechanical wear, servo-motor effects, or operational delays, was observed, reflecting the naturally coupled mechanical–rheological nature of real industrial data.

The proposed solution offers practical advantages for injection moulding operations. Because it relies exclusively on standard machine data, it can be deployed on both modern and legacy systems without additional sensors or hardware modifications. Its ability to anticipate instability minutes in advance provides valuable support for proactive process correction, enabling reductions in scrap rate, improved stability, and more energy-efficient production. Moreover, its responsiveness to both melt behaviour and machine-mechanical signatures highlights additional potential for integration into predictive-maintenance workflows.

Future work will focus on validating the proposed framework across multiple polymer materials, moulds, and injection moulding machines operating under different process conditions. In addition, further research will explore physics-informed modelling constraints, transformer-based architectures, and autoencoder-derived representations to improve robustness and interpretability, particularly in disentangling rheological behaviour from machine-induced variability. Enhanced multimodal sensing, such as thermography or acoustic monitoring, may further strengthen the link between learned features and polymer-processing physics. Finally, integrating the predictive model into adaptive or closed-loop control strategies could enable fully self-regulating melt-stabilisation systems, advancing injection moulding toward Industry 4.0’s vision of intelligent, autonomous manufacturing.

## Figures and Tables

**Table 1:** Double Torque features (2F), predicting next time step.

Class	Precision	Recall	F1-Score	Support
Normal (0)	0.96	0.96	0.96	1275
Anomaly (1)	0.91	0.91	0.91	512
Accuracy			0.95	1787
Macro Avg	0.94	0.94	0.94	1787
Weighted Avg	0.95	0.95	0.95	1787

**Table 2:** Explained Variance features (33F), predicting next time step.

Class	Precision	Recall	F1-Score	Support
Normal (0)	0.96	0.96	0.96	1275
Anomaly (1)	0.91	0.91	0.91	512
Accuracy			0.95	1787
Macro Avg	0.94	0.94	0.94	1787
Weighted Avg	0.95	0.95	0.95	1787

**Table 3:** Pearson Correlation features (14F), predicting next time step.

Class	Precision	Recall	F1-Score	Support
Normal (0)	0.96	0.96	0.96	1272
Anomaly (1)	0.90	0.90	0.90	505
Accuracy			0.94	1777
Macro Avg	0.93	0.93	0.93	1777
Weighted Avg	0.94	0.94	0.94	1777

**Table 4:** Double Torque features (2F), predicting defect within next 7 steps.

Class	Precision	Recall	F1-Score	Support
Normal (0)	0.84	0.88	0.86	1055
Anomaly (1)	0.81	0.76	0.79	731
Accuracy			0.83	1786
Macro Avg	0.83	0.82	0.82	1786
Weighted Avg	0.83	0.83	0.83	1786

**Table 5:** Pearson Correlation features (14F), predicting defect within next 7 steps.

Class	Precision	Recall	F1-Score	Support
Normal (0)	0.82	0.92	0.86	1053
Anomaly (1)	0.85	0.70	0.77	723
Accuracy			0.83	1776
Macro Avg	0.84	0.81	0.82	1776
Weighted Avg	0.83	0.83	0.83	1776

**Table 6:** Explained Variance features (33F), predicting defect within next 7 steps.

Class	Precision	Recall	F1-Score	Support
Normal (0)	0.81	0.93	0.86	1055
Anomaly (1)	0.87	0.69	0.77	731
Accuracy			0.83	1786
Macro Avg	0.84	0.81	0.82	1786
Weighted Avg	0.83	0.83	0.82	1786

**Table 7:** Performance summary across feature configurations and horizons.

Feature Set	Horizon	F1-Score	AUC
Double Torque (2)	Next Step	0.94	0.974
Double Torque (2)	Next 7 Steps	0.82	0.91
Pearson Corr. (14)	Next Step	0.93	0.960
Pearson Corr. (14)	Next 7 Steps	0.82	0.89
Explained Var. (33)	Next Step	0.94	0.975
Explained Var. (33)	Next 7 Steps	0.82	0.90

## Data Availability

The original contributions presented in this study are included in the article. Further inquiries can be directed to the corresponding author.
